# Measures of Engagement With mHealth Interventions in Patients With Heart Failure: Scoping Review

**DOI:** 10.2196/35657

**Published:** 2022-08-22

**Authors:** Ifeanyi Madujibeya, Terry Lennie, Adaeze Aroh, Misook L Chung, Debra Moser

**Affiliations:** 1 College of Nursing University of Kentucky Lexington, KY United States; 2 Department of Public Health College of Health Professions Slippery Rock University Slippery Rock, PA United States

**Keywords:** heart failure, mobile health interventions, mHealth interventions, patient engagement, system usage data, heart failure outcomes, mobile phone

## Abstract

**Background:**

Despite the potential of mobile health (mHealth) interventions to facilitate the early detection of signs of heart failure (HF) decompensation and provide personalized management of symptoms, the outcomes of such interventions in patients with HF have been inconsistent. As engagement with mHealth is required for interventions to be effective, poor patient engagement with mHealth interventions may be associated with mixed evidence. It is crucial to understand how engagement with mHealth interventions is measured in patients with HF, and the effects of engagement on HF outcomes.

**Objective:**

In this review, we aimed to describe measures of patient engagement with mHealth interventions and the effects of engagement on HF outcomes.

**Methods:**

We conducted a systematic literature search in 7 databases for relevant studies published in the English language from 2009 to September 2021 and reported the descriptive characteristics of the studies. We used content analysis to identify themes that described patient engagement with mHealth interventions in the qualitative studies included in the review.

**Results:**

We synthesized 32 studies that operationalized engagement with mHealth interventions in 4771 patients with HF (3239/4771, 67.88%, male), ranging from a sample of 7 to 1571 (median 53.3) patients, followed for a median duration of 90 (IQR 45-180) days. Patient engagement with mHealth interventions was measured only quantitatively based on system usage data in 72% (23/32) of the studies, only qualitatively based on data from semistructured interviews and focus groups in 6% (2/32) of studies, and by a combination of both quantitative and qualitative data in 22% (7/32) of studies. System usage data were evaluated using 6 metrics of engagement: number of physiological parameters transmitted (19/30, 63% studies), number of HF questionnaires completed (2/30, 7% studies), number of log-ins (4/30, 13% studies), number of SMS text message responses (1/30, 3% studies), time spent (5/30, 17% studies), and the number of features accessed and screen viewed (4/30, 13% studies). There was a lack of consistency in how the system usage metrics were reported across studies. In total, 80% of the studies reported only descriptive characteristics of system usage data. The emotional, cognitive, and behavioral domains of patient engagement were identified through qualitative studies. Patient engagement levels ranged from 45% to 100% and decreased over time. The effects of engagement on HF knowledge, self-care, exercise adherence, and HF hospitalization were inconclusive.

**Conclusions:**

The measures of patient engagement with mHealth interventions in patients with HF are underreported and lack consistency. The application of inferential analytical methods to engagement data is extremely limited. There is a need for a working group on mHealth that may consolidate the previous operational definitions of patient engagement into an optimal and standardized measure.

## Introduction

### Background

Heart failure (HF) is a progressive chronic health condition characterized by the inability of the heart muscle to pump sufficient blood to meet the metabolic demands of the body [1]. HF is characterized by a high incidence of acute exacerbations, leading to poor health-related quality of life, and high hospitalization and mortality rates [2]. An estimated 6.2 million adults aged 20 years and older have HF in the United States [2]. This prevalence rate is projected to increase by 46% by 2030 [2].

Mobile devices are increasingly leveraged in mobile health (mHealth) interventions to provide comprehensive and personalized care that may decrease the incidence of HF exacerbations, improve health-related quality of life, and decrease HF hospitalization and mortality rates. mHealth is the use of mobile devices, such as smartphones, wearable sensors, PDAs, tablet computers, and mobile telemonitoring devices to deliver care, maintain health, and manage chronic conditions [3-5]. The outcomes of mHealth interventions for patients with HF have been inconsistent. Previous meta-analyses [6,7] and a systematic review [8] of mHealth-based interventions have shown mixed evidence on the effectiveness of these interventions in improving outcomes in patients with HF. Considering that engagement with mHealth interventions is a prerequisite for the effectiveness of the interventions [9], poor patient engagement with the interventions might be associated with mixed results [10-14]. Hence, it is crucial to measure engagement with mHealth interventions in patients with HF.

### Conceptualization of Patient Engagement With mHealth

On the basis of an expert consensus, Yardley et al [9] conceptualized engagement with mHealth interventions as a dynamic process involving microengagement and macroengagement. Microengagement is the moment-to-moment use of mHealth interventions or systems and the subjective experience that is derived from using the systems. Macroengagement is the degree of health-related behavior change that is mediated by the use of mHealth interventions [9]. Perski et al [15] extended the framework proposed by Yardley et al [9] by describing subjective user experience as attention, interest, and affect [10,15]. Hence, patient engagement with mHealth was operationalized in previous studies as the intensity, duration, and frequency of mHealth system use [10,15-18], as well as the subjective experiences of the users, defined as attention, interest, and affect [15].

Short et al [11] advanced previous work [9,15] by identifying 8 subthemes that may be used in qualitative research to describe subjective user experience (Multimedia Appendix 1). Accordingly, we conceptualized engagement with mHealth interventions as a dynamic and multidimensional construct that consists of behavioral, cognitive, and emotional domains. The behavioral domain is measured using system usage data, which are quantitative data generated by the physical interaction of a user with mHealth systems [9,11]. Cognitive (pertains to what a patient thinks or knows) and emotional (what a patient feels) domains describe subjective user experiences of using mHealth [11,15,19].

### Gap in Evidence

There is a dearth of information on how engagement with mHealth interventions has been conceptualized and measured in patients with HF. Recent scoping reviews [10,20] of evidence on measures of engagement in mHealth interventions for the management of chronic conditions included 51 articles in which patient engagement measures were reported. Only 3 articles were reviewed related to patients with HF. However, 3 previous reviews of mHealth applications for HF self-care [21-23] identified 70 available mHealth applications for the management of HF. Patient engagement with these applications has not been reported. Thus, previous scoping reviews [10,20] might not be a full representation of current mHealth engagement research in patients with HF.

In addition, previous scoping reviews [10,20] focused only on system usage data, which is an objective measure of usage logs generated during a user’s interaction with mHealth systems. System usage data may not capture subjective user experiences, which are an essential aspect of patient engagement with mHealth interventions [9,11,17]. Thus, a review that includes both objective and subjective measures of patient engagement with mHealth is warranted. This review aimed to synthesize current evidence on measures of engagement of patients with HF with mHealth interventions and examine the effects of patient engagement with mHealth interventions on HF outcomes.

Specifically, we addressed the following questions: (1) How was engagement with mHealth interventions operationalized quantitatively and qualitatively in patients with HF? (2) How was engagement with mHealth interventions in patients with HF analyzed and reported in previous studies in patients with HF? (3) What were the patterns of engagement over time? (4) What factors predicted patterns of engagement over time? (5) What was the relationship between engagement and HF outcomes?

## Methods

### Methodological Framework

As a result of the novelty and heterogeneity of mHealth interventions in patients with HF [6,8], we used a scoping review to synthesize current evidence on engagement with mHealth interventions in patients with HF [24-28]. The review followed the checklist of the PRISMA-ScR (Preferred Reporting Items for Systematic Reviews and Meta-Analyses extension for Scoping Reviews) [25]. The review was guided by the 6-step methodological framework for scoping reviews by Arksey and O’Malley [28-30] except for the optional consultation phase (step 6) [26]. The 5 steps used in this review are as follows: (1) formulate a research question; (2) search the literature to identify relevant studies; (3) select the relevant studies based on predefined eligibility criteria; (4) chart the data to identify key information; and (5) organize, summarize, and report the findings [27-30].

We conducted a comprehensive search of the literature in 7 databases (CINAHL, Cochrane Central Register of Controlled Trials [CENTRAL], PubMed, Scopus, PsycINFO, MEDLINE, and Ovid) for relevant literature published in the English language from 2009 to September 2021. The search was conducted with the help of an experienced medical librarian. A combination of keywords was used to search the databases (Multimedia Appendix 2).

### Eligibility Criteria

The following were the inclusion criteria: (1) studies that included adult patients with HF, aged ≥18 years, in New York Heart Association class 1 to 4, of any sex, ethnicity, and nationality, and published in the English language between 2009 and September 2021; (2) studies that operationalized engagement with mHealth interventions or usage of mHealth systems; and (3) studies that included results of patient engagement with mHealth interventions or effects of engagement with mHealth interventions on patient outcomes. Usability and feasibility studies in which patients explored mHealth application features only once were excluded because one-time usage is insufficient to establish patient engagement with the intervention [10]. Landline telephone–based interventions were also excluded because landline telephones are not considered mobile devices.

### Data Extraction and Analysis

**Figure 1:**
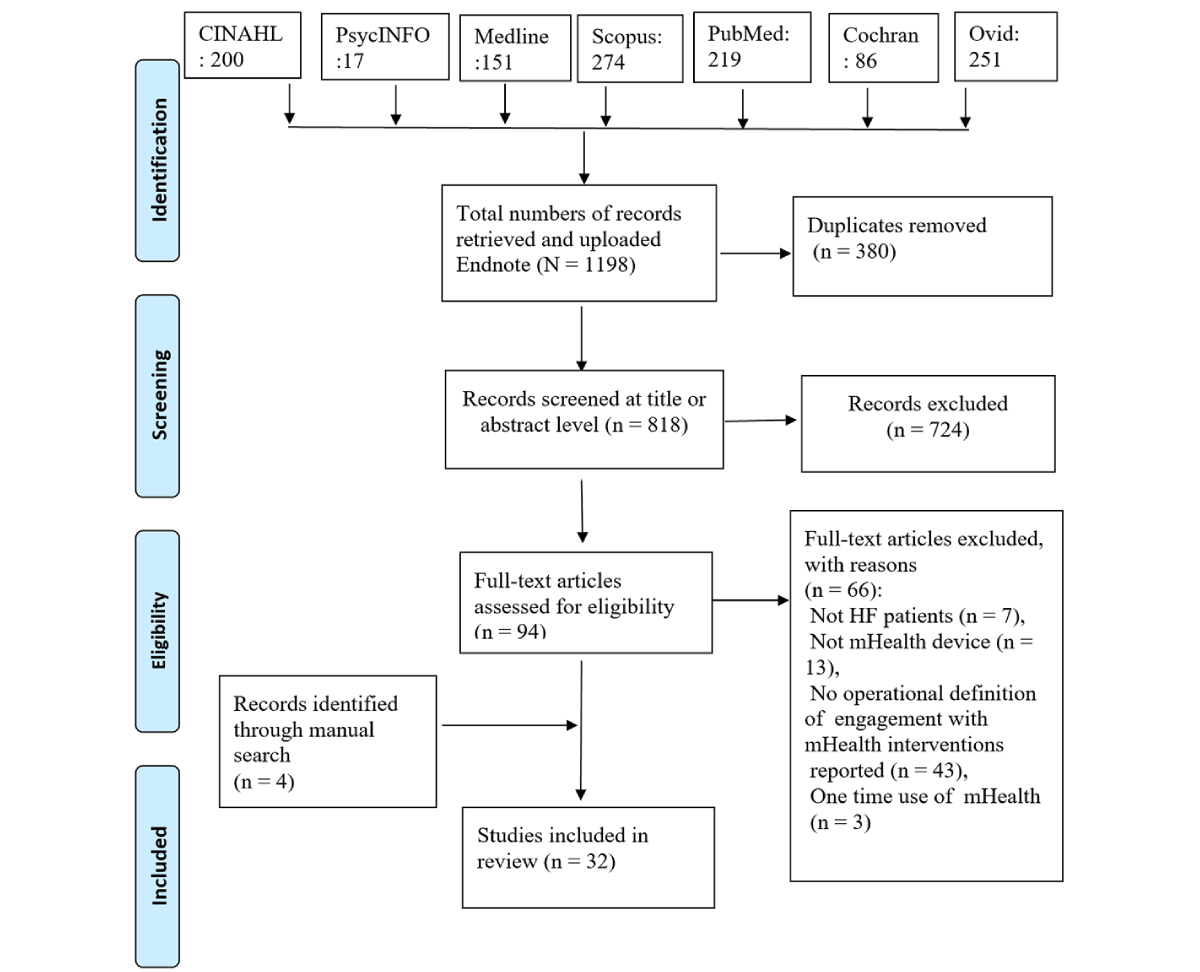
Preferred Reporting Items for Systematic Reviews and Meta-Analyses flow diagram.

The initial database search yielded 1198 articles. The articles were uploaded to the Endnote software (version 20) for analysis. The selection process is illustrated in the PRISMA (Preferred Reporting Items for Systematic Reviews and Meta-Analyses) flow diagram (Figure 1). Two reviewers (first and third authors) independently selected 32 studies from the 1198 that met the inclusion criteria. The study and intervention characteristics were coded using a data extraction form based on related constructs from the CONSORT‐EHEALTH (Consolidated Standards of Reporting Trials of Electronic and Mobile Health Applications and Online Telehealth) checklist (V.1.6.1) [31]. The coded characteristics are presented in Textbox 1. The descriptive characteristics of the studies were reported. System usage data reported in the studies were categorized using the frequency, intensity, time, and type (FITT) principle to provide more insight into the usage data. The FITT principle has been previously described by Short et al [11] and applied in analyzing the system usage data [32]. Frequency describes how often a patient completes a required task. The intensity or depth is the proportion of an assigned task completed by a patient. Time measures the duration for completing a task, and type is attributed to the type of intervention [11].

All studies in which qualitative methods were used to measure patient engagement were uploaded to the qualitative data analysis software Atlas.ti (version 8). The 3 phases of deductive content analysis outlined by Elo et al [33] were used to analyze the qualitative data. In the first phase, line-by-line coding was performed by grouping the data into clusters of information and assigning labels to the clusters. In the second phase, the list of codes was combined into potential subthemes and themes in accordance with the 8 main constructs used by Short et al [11] to describe the emotional, cognitive, and behavioral domains of engagement. Although the constructs overlapped, Short et al [11] provided a concise description of each construct (Multimedia Appendix 2). In the third phase, the potential themes and subthemes were refined to ensure that the data within each theme were distinctive. Two authors (IM and AA) independently conducted the initial analysis, which was reviewed by all the coauthors. Any disagreement during the analytical process was discussed until a consensus was reached.

**Codes extracted from included studies.**

**Study characteristics**

- General information about the studies including the first author’s last name, year, country of publication, duration of follow-up, patient characteristics, and the purpose of the study

**Mobile health (mHealth) characteristics**

- mHealth devices: mobile phone, PDA devices, sensor, and mobile telemonitor systems
- Measured physiological parameters: blood pressure, heart rate, weight, oxygen saturation, and electrocardiogram transmitted by patients
- Data transmission: mode of transmitting data from peripheral devices, such as weighing scale to the mHealth device
- Transmission frequency: how often patients transmit physiological parameters to providers or central monitoring centers
- The interactive user interface: interface for patient’s interaction with mHealth systems

**Engagement measures**

- Operationalization of engagement: how engagement was measured
- Objective measures: objective measures of engagement, such as quantitative measures of system usage
- Subjective measures: measures of engagement using self-reported questionnaires or through a qualitative method, such as interviews
- Data collection method: methods for collecting engagement information
- Analytical methods: methods used for analyzing engagement data
- Reported engagement: the results of engagement with mHealth
- Effect of engagement: the reported effects of engagement on patient-reported outcomes.
- The strengths and limitations of studies

## Results

### Study and Patient Characteristics

Of the 32 studies, 16 (50%) [14,34-48] were conducted in the United States. The remaining studies were conducted in Germany (3/32, 9%) [49-51], Canada (2/32, 6%) [14,52], Belgium (2/32, 6%) [53,54], Italy (2/32, 6%) [55,56], the United Kingdom (1/32, 3%) [57], Austria (1/32, 3%) [58], Sweden (1/32, 3%) [59], Poland (1/32, 3%) [60], Singapore (1/32, 3%) [61], China (1/32, 3%) [62], and Australia (1/32, 3%) [63]. The duration of the studies ranged from 1 to 26 months, with a median of 3 months. The sample sizes ranged from 7 to 1571, with a median of 53.3 patients. Most of the patients (3239/4771, 67.9%) were male.

### Intervention Characteristics

The key characteristics of the interventions are presented in (Multimedia Appendix 3). In approximately 50% (16/32) of the studies [14,35,38,40,43,45,47-49,52-55,57,58,60], patients used smartphones, 28% (9/32) [36,37,39,42,46,59,61-63] used tablet computers, 6.25% (2/32) [50,56] used PDAs, and 16% (5/32) used either portable telemonitoring devices [34,44] or a combination of smartphones, smart watches, and tablet computers [45,51,64] as integral components of mHealth systems for the management of HF symptoms or for the provision of HF-related self-care education.

In 84% (27/32) of the studies, physiological parameters (weight, blood pressure, heart rate, or electrocardiogram), patient-reported HF symptoms, and self-care activities that were transmitted to either secured servers or telemonitoring centers were used to provide personalized HF remote monitoring and management. In 9% (3/32) of the studies, the mHealth intervention focused solely on providing HF-related self-care education through daily HF quizzes [34], game application [37], or daily SMS text messages [35]. In the remaining 6% (2/32) of the studies [60,64] investigators used mHealth systems to implement home-based cardiac rehabilitation or to target exercise adherence via videoconferencing. The investigators in 91% (29/32) of the studies incorporated the user interface of the mHealth devices to provide interactive HF education, graphic display of monitored parameters, activity reminders, or interaction with a web-based assistant.

### Operational Definitions of Patient Engagement With mHealth

In addition, the operational definitions of patient engagement with mHealth interventions are summarized in Multimedia Appendix 4. Patient engagement with mHealth interventions was measured solely based on system usage data in 72% (23/32) of the studies. Among the remaining studies, 6% (2/32) used only qualitative methods to determine engagement (focus groups and semistructured interviews) [34,39]; 19% (6/32) [14,40,48,54,57,62] used both system usage data and qualitative methods; and 3% (1/32) [43] planned to use system usage data, qualitative methods (think aloud), and user engagement questionnaires.

### Analytical Methods Applied to System Usage Data

As shown in Multimedia Appendix 5, in 94% (30/32) of the reviewed studies, patient engagement with mHealth interventions was evaluated using six main system usage data: (1) number of physiological parameters measured and transmitted (19/30, 63%), (2) number of HF symptom questionnaires completed (2/30, 7%), (3) number of log-ins (4/30, 13%), (4) number of SMS text message responses (1/30, 3%), (5) time spent (5/30, 17%), and (6) number of features accessed or screens viewed (4/30, 13%). Descriptive statistics (mean, range, median, and percentage) were used to summarize patient engagement in 80% (24/30) of the reviewed studies that analyzed system usage data. The remaining 20% (6/30) of the studies applied both descriptive and inferential statistics to system usage data. The analytical methods and studies that used them are presented in Multimedia Appendix 4.

### Operational Definitions of System Usage Data

Operational definitions of system usage data and reported outcomes are presented in Multimedia Appendices 4 and 6. The operational definitions differed across the 30 studies that reported the metric. In 47% (14/30) of the 30 studies, system usage data were operationalized as the proportion of patients who used an mHealth system to complete 70% [49,50], 80% [52], 85% [61], or 100% [36,38,40,42,48,60-64] of the required tasks as expected during the duration of intervention or system use. The engagement levels reported in the 14 studies ranged from 45% [63] to 100% [60]. In 23% (7/30) of the 30 studies [14,40,41,46,53,57,59], system usage data were measured as the proportion of days during which patients completed assigned tasks or used mHealth, as expected, during the total number of days equipped with the system. Median engagement rates of 88% and 96% were reported in 2 studies [41,59], while 1 study [40] reported a mean engagement of 18.2%. The remaining 4 studies [14,46,53,57] reported engagement outcomes as a percentage, ranging from 73.6% [46] to 88% [57].

In 20% (6/30) of the 30 studies [35,44,52,54,55,58], system usage data were operationalized as the number of assigned tasks completed per patient per number of days equipped with a mobile device or intervention. In 1 of the 6 studies [35], investigators reported a mean engagement of 5.7%, with a range of 0 to 27, while in the remaining studies, engagement was reported as an overall rate, ranging from 53.3% to 95%. In 7% (2/30) of the studies [37,51], investigators measured system usage data as the number of times an mHealth system was used per patient per duration of intervention. The reported engagement ranged from 9.7 hours in 28 days [37], to 11.3 hours in 60 days [51]. Other investigators [45,47] measured system usage data as the ratio of the number of hours a patient had heart rate readings to the total hours in the study. For example, Sohn et al [45] reported an engagement rate of 79.1%.

The categorization of system usage data based on the FITT principle is presented in Table 1. The intensity category was the most predominant (22/30, 73.3%) among the reviewed studies, followed by frequency (12/30, 40%), and time (8/30, 26.7%). Only 2 studies [40,48] reported the frequency, intensity, and time spent.

**Table 1:** Categorization of system usage data based on the frequency, intensity, time, and type (FITT) principle.

Study	mHealth^a^ device	Frequency	Intensity	Time spent	Type of intervention
Apergi et al [46]	Tablet	N/A^b^	✓	N/A	Telemonitoring
Athilingam et al [38]	Smartphone	N/A	✓	✓	Telemonitoring, HF^c^ education, and physical activity
Bartlett et al [57]	Smartphone	✓	N/A	✓	Telemonitoring, physical activity, and HF education
Buck et al [39]	Tablet	N/A	N/A	N/A	Telemonitoring and physical activity
Chow et al [61]	Tablet	N/A	✓	N/A	Telemonitoring and HF education
Dang et al [40]	Smartphone	✓	✓	✓	Telemonitoring
Deka et al [64]	Smartwatch	N/A	✓	N/A	Physical activity
Dendale et al [53]	Smartphone	N/A	✓	N/A	Telemonitoring
Ding et al [63]	Tablet	✓	N/A	N/A	Telemonitoring
Guo et al [62]	Tablet	✓	N/A	N/A	Telemonitoring
Hägglund et al [59]	Tablet	✓	N/A	N/A	Telemonitoring and HF education
Hayes et al [44]	Tablet, WTD^d^	✓	✓	N/A	Telemonitoring and HF education
Kitsiou et al [47]	Smartphone, smartwatch	N/A	✓	✓	Telemonitoring and physical activity
Koehler et al [49]	WTD, smartphone, or tablet,	N/A	✓	N/A	Telemonitoring and HF^a^ education
Koehler et al [50]	PDA	N/A	✓	N/A	Telemonitoring
Lloyd et al [42]	Tablet	✓	✓	N/A	Self-care and physical activity
Louise et al [34]	WTD	N/A	N/A	N/A	HF education
Nundy et al [35]	Smartphone	✓	N/A	N/A	HF education
Piotrowicz et al [60]	Smartphone	N/A	✓	N/A	Cardiac rehab and HF education
Pedone et al [55]	Smartphone	N/A	✓	N/A	Telemonitoring
Radhakrishnan et al [37]	Tablet	N/A	✓	✓	HF education via gaming
Rosen et al [41]	Tablet	N/A	✓	N/A	Telemonitoring and HF education
Scherr et al [58]	Smartphone	✓	N/A	N/A	Telemonitoring
Seto et al [52]	Smartphone	N/A	✓	N/A	Telemonitoring
Smeets et al [54]	Smartphone	N/A	✓	N/A	Telemonitoring and HF education
Sohn et al [45]	Smartwatch, smartphone	N/A	✓	✓	Telemonitoring and physical activity
Villani et al [56]	PDA	N/A	✓	N/A	Telemonitoring
Ware et al [14]	Smartphones	N/A	✓	N/A	Telemonitoring
Wei et al [48]	Smartphones	✓	✓	✓	Telemonitoring and HF education
Werhahn et al [51]	Smartphones, tablet smartwatch	✓	N/A	✓	Telemonitoring and physical activity
Zan et al [36]	Tablet, web portal	N/A	✓	N/A	Telemonitoring
Zhang et al [43]	Smartphones with virtual reality–based self-care assistance	N/A	N/A	N/A	Telemonitoring and physical activity

^a^mHealth: mobile health.

^b^N/A: not applicable; represents qualitative studies or studies that did not report elements of the FITT principle.

^c^HF: heart failure.

^d^WTD: wireless telemonitoring device.

### Longitudinal Patterns of Patient Engagement With mHealth Interventions

The investigators in one of the 8 studies [41] that reported longitudinal patterns of patient engagement with mHealth interventions concluded that patient engagement did not change over time. However, the investigators did not state how the effect of time on patient engagement patterns was examined. In the remaining 7 studies, the investigators used descriptive statistics (plots of engagement over time) [36,40,42,44,46,52,62] or a longitudinal analysis [14] to examine the effects of time on engagement patterns. All the investigators reported that patient engagement decreased over time.

### Predictors of Patient Engagement With mHealth

Four groups [14,41,45,46] examined the effects of age on patient engagement with mHealth interventions, and the findings were inconclusive. Apergi et al [46] reported a positive association between age and patient engagement, whereas Sohen et al [45] and Rosen et al [41] reported a nonsignificant association between age and patient engagement. The investigators in 2 studies [14,41] examined the effects of sex and HF severity (measured by New York Heart Association class) on patient engagement. They reported a nonsignificant association among gender, HF severity, and patient engagement with mHealth interventions.

### Qualitative Measures of Patient Engagement

The emotional, cognitive, and behavioral domains of patient engagement with mHealth interventions, and the constructs used to describe them in qualitative research are summarized in Table 2. In 8 [14,34,39,40,48,54,57,62] out of the 9 studies that included qualitative measures, open-ended questionnaires, focus groups, and semistructured interviews were used to describe patients’ experience of using mHealth devices. The experiences were categorized under the behavioral, cognitive, and emotional domains of patient engagement. Intervention usage, which is a construct of the behavioral domain that describes a user’s patterns of interaction with mHealth interventions or systems [11,19], was the most reported subcategory (7/8, 88%) in the studies. For example, in the postintervention interviews with patients who participated in a tablet-delivered self-care intervention (Penn State Heart Assistant), patients stated that they recorded their blood pressure and weight every morning and exercised daily whenever the mHealth system was functional [39].

Three [14,40,62] out of the 8 studies used affect to describe the emotional domain of patient engagement. For example, in a mobile phone–based telemonitoring intervention, patients stated that they felt guilty when they missed measuring the required daily physiological parameters [14]. In 11.1% (1/9) [43] of the studies that included qualitative measures, investigators planned to use think aloud to capture the patient’s cognitive process while patients were performing tasks on mHealth applications.

**Table 2:** Qualitative constructs used to describe the emotional, cognitive, and behavioral engagement.

Study	Subcategories	Quotes
Barlett et al [57]	Intervention usage^a^ Immersion^b^	“The interview data report higher engagement with the walking than was recorded in the step count in the mobile device.” (Intervention usage) “I cannot use the system every day, I will use it as it fit my lifestyle.” (Immersion)
Buck et al [39]	Intervention usage^a^	“I still record my blood pressure, weight, and exercise every day. So, instead of a paper, I would put it on my iPad.” (Intervention usage)
Dang et al [40]	Affect^c^ Intervention usage^a^	“All participants said that the program made them feel more secure about their health and that they would stay enrolled.” (Affect) “Since participants received daily reminders to weigh themselves, it had become a habit.” (Intervention usage)
Guo et al [62]	Interest^c^ Affect^c^	Participants were more interested in smart health tracking devices, which could help them keep track of health conditions anywhere, (interest) so that they felt more secure and involved in their care (affect)
Laframboise et al [34]	Intervention usage^a^ Interest^b^	“Many participants perceived the daily interaction with the Health Buddy (mobile device) as social contact and something they looked forward to, as well as something to do daily.” (Interest) “The Health Buddy was kind of like a good friend. It gave me something to do every day.” (Intervention usage and interest)
Smeets et al [54]	Intervention usage^a^	“50% of patients were eager to continue using the CardioCoach follow-up tool after the study ended.” (Intervention usage)
Ware et al [14]	Intervention usage^a^ Affect^c^ Interest^b^	“Taking my readings is what I do first thing in the morning before I get the phone call with the annoying ringing” (Intervention usage, affect) “Feel kind of guilty because I haven’t got it [Taking daily readings] done.” (Affect)
Wei et al [48]	Intervention usage^a^	“One participant reported synching issues between the scale and the app.” (Intervention usage)

^a^Behavioral domain.

^b^Cognitive domain.

^c^Emotional domain.

### Effects of Patient Engagement With mHealth Interventions on HF Outcomes

Few researchers reported the effect of patient engagement with mHealth interventions on HF outcomes (HF knowledge, self-care, weight loss, and exercise engagement) using both quantitative and qualitative methods. Patient engagement with mHealth interventions was positively correlated with an improvement in HF knowledge. Three studies aimed to improve HF knowledge using daily HF quizzes [57], mobile game applications [37], or watching HF educational videos on smartphone interfaces [48]. There was a significant positive correlation between patient engagement and improvement in HF knowledge in all 3 studies.

Radhakrishnan et al [37] reported a positive correlation between the average game-playing time and HF-related self-care. In contrast, Sohn et al [45] showed a negative correlation between patient engagement and self-care confidence*.* In the 3 studies in which semistructured interviews were used [14,34,40], patients stated that engagement in telemonitoring was associated with improvement in their HF self-care [14,34] and self-care confidence [40]. However, only 33% (8/24) of the patients interviewed in one study [14] agreed that engagement with the intervention improved their self-care confidence. Thus, based on qualitative data, the effect of patient engagement on self-care is inconclusive.

Only one investigative team [42] examined the effects of patient engagement with interventions on weight loss and exercise. The investigators reported positive associations between patient engagement, weight, and exercise engagement. On the other hand, only Haynes et al [44] examined the effect of patient engagement on hospitalization because of HF. They reported that every 1-day increase in patient engagement was associated with a 19% decrease in HF hospitalization [44].

## Discussion

### Principal Findings

We used a scoping review to present an overview of how engagement with mHealth interventions was operationalized among patients with HF. Across the studies, patient engagement with the interventions was evaluated using both quantitative measures based on system usage data and qualitative measures based on semistructured interviews and focus groups. System usage data were evaluated as physiological parameters transmitted to telemonitoring centers, number of HF questionnaires completed, number of log-ins, number of SMS text message responses, time spent engaging with interventional features, features accessed, or screen viewed. The measures of system usage data were underreported and lacked consistency. The application of inferential analytical methods to the data is extremely limited.

### Evaluation of System Usage Data

In most studies in our review (23/32, 72%), only system usage data were measured to quantify engagement with mHealth. The predominant focus on system usage data in the reviewed studies was expected, considering that these metrics are the most reported measures of patient engagement with mHealth interventions [10,11,15]. mHealth devices can automatically track the user’s patterns of interaction with mHealth interventions and generate quantitative data that reflect the patterns of the interaction. The ready availability of the data may have contributed to its popularity among investigators. However, this method alone misses important components of engagement.

Reporting all 4 main elements of the FITT principle is essential to capture all the aspects of system usage data [11,65-67]. However, only 2 studies in our review reported all 4 components of the FITT intervention. In 47% (14/30) of the studies that evaluated system usage data, investigators reported only intensity. The emphasis on intensity was consistent with previous studies [10,15] that categorized system usage metrics as amount, breadth, duration, and depth. Pham et al [10] reported that the majority (31/41, 76%) of the studies in their review measured the depth of engagement category, which is the same as the intensity [11]. It is likely that the investigators were not examining the frequency and time components of the FITT principle or were underreporting them. This could obscure the differences in patient engagement profiles when patients showed similar intensity levels, but differed in either frequency or time spent in mHealth interventions. Examining all components of FITT is essential in gaining more insight into patient engagement behaviors than measuring only one component. Such insight could guide actions and policies to promote engagement behaviors that are congruent with interventional outcomes [11].

### Longitudinal Patterns of Patient Engagement

Cheikh-Moussa et al [20] concluded in their review of 10 articles that patients with cardiometabolic conditions’ engagement with mHealth interventions decreased over time. The findings are consistent with the results from 8 articles in our review that showed that patient engagement with mHealth interventions decreased over time. However, our findings should be interpreted with caution. The investigators in 7 of the 8 studies used only simple plots (descriptive statistics) to examine the relationship between patient engagement and time. Similarly, researchers in 2 studies [41,45] out of the 4 that examined the effect of age on engagement limited their analysis to descriptive statistics. Thus, the application of inferential statistics in evaluating system usage data is extremely limited, making it challenging to draw definitive conclusions on the longitudinal patterns of patient engagement and the predictors of patient engagement with mHealth interventions.

### Subjective Measures of Engagement

Intervention usage was the most identified qualitative measure of patient engagement, indicating that most investigators focused on usage (behavioral domain). These findings appear consistent with a previous qualitative review of 11 studies that evaluated patient engagement with eHealth [19]. The investigators highlighted the behavioral and cognitive domains of engagement as the most assessed aspects of patient engagement [19]. However, the emotional domain is equally important in understanding the complexity of patient engagement with mHealth interventions. For example, the experience of technical challenges with mHealth interventions could trigger negative emotions in patients, such as emotional exhaustion and sadness. Patients may be inclined to regulate these emotions by decreasing the extent of their interaction with the intervention. Hence, the interplay between the emotional and behavioral domains of engagement within the context of technical problems could influence patterns of patient engagement with mHealth [68]. Thus, assessing the 3 domains of patient engagement may be pivotal in understanding the complexity of patient engagement with mHealth interventions.

The qualitative assessment of intervention usage may be combined with system usage data to provide more insight into the patterns of patient engagement with mHealth interventions. For example, in 2 studies, the SMART Personalized Self-management System for HF intervention [57] and phone-based telemonitoring intervention for patients with HF [14], the investigators deduced from interview reports that system usage data captured by the mHealth system did not reflect the actual patient engagement. The patients reported a higher degree of engagement, but it was not captured by the mHealth systems because of technical problems such as poor connectivity between peripheral devices and mobile phones, server downtime, and system malfunction [57]. Thus, the use of both qualitative and quantitative approaches to measure patient engagement with mHealth is recommended.

Focusing only on the qualitative method may present an inaccurate representation of patient engagement, considering that the findings of qualitative methods are subject to social desirability and recall bias. For example, in the Health Buddy intervention [34], the interview was conducted approximately 2 years after the intervention was completed. However, the patients may not recall their experiences of using the intervention. Thus, both system usage data and qualitative methods have limitations that may hamper the accurate capture of patient engagement data. However, both methods may complement each other when combined.

### Effects of Engagement on HF Outcomes

We determined that the effects of patient engagement on HF outcomes were inconclusive owing to the lack of rigorous analytical methods in the reviewed studies. For example, in 3 studies [37,48,57] that examined the relationship between patient engagement and HF knowledge, only correlation analyses were used. Correlation analysis can be used to summarize sample characteristics, but an inferential analytical approach is essential for making an inference about a population from a sample. The effects of patient engagement on weight loss, exercise, and HF hospitalizations were examined in only 1 study. Although the findings were promising, there is insufficient evidence to conclude that patient engagement with mHealth is associated with improvements in HF outcomes [11].

### Study Implications

The CONSORT-EHEALTH checklist for reporting eHealth and mHealth interventions highly recommends reporting operational definitions of patient engagement [31]. The findings from our study and previous reviews [10,20,69,70] indicate the lack of a standard approach for measuring patient engagement with mHealth interventions. Across studies, different cutoff points were used to indicate effective patient engagement, without any supporting evidence for choosing the cutoff points. To ensure the comparison of findings across studies, addressing the inconsistency in measures of patient engagement should be a key research priority.

International working groups on mHealth have been previously used to develop strategies and policies to support the global implementation of effective mHealth initiatives [71], and unify previous conceptual definitions of patient engagement into an integrative definition of patient engagement [72,73]. Thus, a working group on mHealth could be established to consolidate previous operational definitions of patient engagement into a standardized measure and determine a cutoff point for effective engagement that could be applied across studies. Moreover, when possible, validated self-reported questionnaires of patient engagement with mHealth, such as the Digital Behavior Change Interventions scale [17] and User Engagement Scale [74,75], may be integrated into mHealth interventions in patients with HF to enable comparison of findings across studies [9].

In 80% (24/30) of studies in which system usage data were analyzed, only descriptive statistics were reported as engagement outcomes. Although patient engagement is conceptualized as a dynamic process that changes over time [9,15,44,76], only 3 studies [14,42,44] in our review applied a longitudinal analytical method to analyze system usage data. The application of longitudinal methods in examining system usage may offer an understanding of how patient engagement with mHealth interventions changes within a person over time, and the effects of the interventions on HF outcomes. Thus, future longitudinal studies with methodological rigor are essential to understand the relationship between patient engagement and HF outcomes and the predictors that influence engagement.

Contemporary mobile devices are embedded with third-party analytical applications, such as Google Analytics [77], Amazon Mobile Analytics [78], Android’s UsageStatsManager [79], and Apple’s Use Screen Time [80]. These applications can capture real-time patterns of patient engagement with mHealth interventions. Surprisingly, only 2 studies in our review used third-party analytical tools to capture patient engagement data. A previous review attributed the minimal usage of analytical applications to investigators’ lack of knowledge of how to extract engagement data from the application [10]. Hence, future investigators should consider collaborating with software developers to design effective approaches for using analytical applications to understand patients’ patterns of engagement with mHealth interventions.

### Strengths and Limitations of the Study

To the best of our knowledge, this is the first scoping review to focus on engagement with mHealth interventions in patients with HF. Unlike previous studies that focused only on quantitative measures [10,69], our review included both objective and subjective measures to capture the wide range of methods that have been used to measure engagement in mHealth interventions among patients with HF.

Our study had some limitations. There was a paucity of studies that examined the relationship between patient engagement with mHealth intervention and HF outcomes, making it challenging to draw conclusions on the effect of the engagement on HF outcomes. The limited number of studies may be related to the small number of articles (N=32) included in our review. The focus of the review on only patients with HF may account for the small number of studies, as mHealth interventions in patients with HF is still at an early stage [8]. Therefore, we conducted a comprehensive literature search with the help of a medical librarian to ensure that all relevant studies were included in the review.

The use of a standardized method to appraise the quality and methodological rigor of the included studies is optional in a scoping review and may be required when the purpose of a review is to appraise the quality of the existing evidence [25]. Considering that the main objective of the present review was to examine the operational definitions of patient engagement with mHealth interventions, a critical appraisal of the existing evidence was not conducted.

In addition, the lack of consistency in the operational definitions of patient engagement in the reviewed studies made it challenging to compare the engagement levels reported across studies. Thus, only the descriptive characteristics of the engagement outcomes are presented in our findings.

### Conclusions

This review indicates that engagement with mHealth interventions in patients with HF has been measured using both quantitative and qualitative approaches. There was a lack of consistency in how the quantitative data were measured across the reviewed studies, making comparisons across studies difficult. The effect of mHealth interventions on HF-related outcomes was inconclusive, possibly related to the investigators’ use of different and incomplete measures of engagement. More research focusing on developing optimal and standardized measures of patient engagement that may be applied across different study designs is warranted. This will facilitate a deeper understanding of patterns of patient engagement with mHealth interventions that may explain variations in intervention outcomes as well as inform future research and policies regarding mHealth interventions.

## Appendix

Multimedia Appendix 1 Conceptual definitions of behavioral, cognitive, and emotional domains.

Multimedia Appendix 2 Databases search strategies and Boolean operators.

Multimedia Appendix 3 The characteristics of mobile health interventions.

Multimedia Appendix 4 The operational definitions of patient engagement with mobile health interventions.

Multimedia Appendix 5 A summary of descriptive characteristics of mobile health (mHealth) intervention engagement metrics and reported outcomes of patient engagement with mHealth interventions.

Multimedia Appendix 6 Reported outcomes of patient engagement with mobile health interventions.

## Abbreviations

CONSORT-EHEALTH: Consolidated Standards of Reporting Trials of Electronic and Mobile Health Applications and Online Telehealth
FITT: frequency, intensity, time, and type
HF: heart failure
mHealth: mobile health
PRISMA: Preferred Reporting Items for Systematic Reviews and Meta-Analyses
PRISMA-ScR: Preferred Reporting Items for Systematic Reviews and Meta-Analyses extension for Scoping Reviews
