# Supplementary material for: Measures of Engagement With mHealth Interventions in Patients With Heart Failure: Scoping Review
Source: JMIR Mhealth Uhealth. 2022 Aug 22;10(8):e35657. doi: 10.2196/35657 (PMC9446141; doi:10.2196/35657)
Supplement: Multimedia Appendix 1 [file mhealth_v10i8e35657_app1.docx]

Multimedia Appendix 1.Conceptual definitions of behavioral, cognitive, and emotional domains

| Categories | Sub-categories | Conceptual Definition |
| --- | --- | --- |
| Emotional | - Interest | It is a long-lasting preference for content or features of mHealth intervention, or a momentary emotional state derived from a user’s involvement with specific mHealth intervention content or feature that keeps the user engrossed with the system. |
| Cognitive | - Attention | The extent of a user’s awareness or concentration on specific mHealth intervention contents or features at the expense of the user’s other daily activities |
| Emotional | - Affect | It describes the feelings that are invoked from the usage of mHealth intervention. |
| Emotional | - Flow | An optimal state at which a user is deeply absorbed in the mHealth system. It is characterized by enjoyment, distorted time perception, and lack of negative affect. |
| Cognitive | - Cognitive absorption | A state of deep involvement with mHealth intervention but unlike flow includes negative affect. |
| Cognitive | - Immersion | It is a less extreme form of engagement with mHealth intervention in which a user may still have awareness of the surroundings |
| Cognitive | - Presence | It is the psychological state of being there. |
| Behavioral | - Intervention   usage | It describes a user’s action that indicates the extent a user has interacted or involved with a mHealth intervention. |
