# Supplementary material for: Measures of Engagement With mHealth Interventions in Patients With Heart Failure: Scoping Review
Source: JMIR Mhealth Uhealth. 2022 Aug 22;10(8):e35657. doi: 10.2196/35657 (PMC9446141; doi:10.2196/35657)
Supplement: Multimedia Appendix 2 [file mhealth_v10i8e35657_app2.docx]

Multimedia Appendix 2. Databases Search Strategies and Boolean Operators

CINAHL with Full Text MEDLINE and PsycINFO
( ( "Heart Failure" OR "Cardiac Failure" OR "Myocardial Failure" OR "Heart Decompensation" OR "Congested heart failure" OR "Acute heart failure" OR "Chronic heart failure" OR "Systolic heart failure" OR "Diastolic heart failure" ) AND ( "30 days Rehospitalization" OR 30-day AND rehospitalization OR "30 Day re-hospitalization" OR "Rehospitalization" OR "Self-Care" OR "Self-Management" OR "self-management" OR "management" OR "self-care" OR "Quality of life" OR "medication adherence" OR "medication-adherence" OR "self-monitoring" OR "physical functioning" OR "self-efficacy" OR "medication compliance" OR "knowledge of heart failure" OR "health behaviors" OR "satisfaction" OR "self-efficacy" OR "self-maintenance" OR "mortality" OR "Death" ) ) AND ( "Mobile Applications" OR "mobile Apps" OR "mobile applications" OR "portable applications" OR "software" OR "smart phones" OR "Telemedicine" OR "eHealth" OR "mobile phones" OR "smartphone" OR "telemetry" OR "Portable software Apps" OR "Mobile Health" OR "mHealth" OR "smart phones" OR "smart phone" ) AND ( "compliance" OR "adherence" OR "engagement" OR "frequency of use" OR "usage metrics" OR "intervention dose" OR "use measure" OR "heaviness of use" OR "intended doses" OR "system use" OR "task compilation" OR "transmission rate" OR "log-ins rate" OR "pages visited" )
