# Supplementary material for: Measures of Engagement With mHealth Interventions in Patients With Heart Failure: Scoping Review
Source: JMIR Mhealth Uhealth. 2022 Aug 22;10(8):e35657. doi: 10.2196/35657 (PMC9446141; doi:10.2196/35657)
Supplement: Multimedia Appendix 3 [file mhealth_v10i8e35657_app3.docx]

Multimedia Appendix 3. The Characteristics of mHealth interventions

| First Author | mHealth Devices | Measured parameters | Data transmission/frequency | Interactive User Interface features. |
| --- | --- | --- | --- | --- |
| Apergi et al [46]. | Echo Dot device equipped with Alexa+ and tablets equipped with visual Avatar | questionnaire on HF self-care and symptoms, | Voice interface | voice interface administered HF-self-care and symptom questionnaire |
| Athilingam et al [38]. | HeartMapp consisted of an android smartphone with a specialized HF app and wearable sensor (Zephyr Bio Harness) | BP, HR, weight, and activity level, | Bluetooth; daily | Android phone. Provided tailored HF education, personalized alerts, graphic symptoms tracker, exercise, medication tracker, |
| Barlett et al [57]. | HTC smartphone, mobile Wi-Fi hotspot (MiFi device) | BP, weight, activity level | Transmission Control Protocol (TCP)/ Internet Protocol (IP) socket technology. | mobile phone, the input of measured parameters, HF quizzes, graphic display of symptoms |
| Buck et al [49]. | Tablet with internet access, | medication intake, weight, activity level | manual entry, daily | Tablet used to view short HF videos, upload measured parameters to servers, and view graphics display of symptoms |
| Chow et al [61]. | Tablet, Sim-card enabled Philips Motiva telehealth system, | BP, HR, and weight | Bluetooth; daily | Tablet computer provided personalized HF self-care education |
| Dang et al [40]. | Smartphone with specialized HF symptoms monitoring application | Weight, patient-reported symptoms | Manual entry; daily | Mobile phone provided daily symptoms questionnaire, and feedback on monitored physiological parameters and symptoms using SMS |
| Dendale et al [53]. | Smartphone | Weight, BP, and HR | Bluetooth; daily | None |
| Deka et al [64]. | Video conferencing application, Fitbit activity monitor | exercise and video conferencing sessions. | Fitbit sensors, manually via exercise diary: N/A | Fitbit provided feedback on activity and heart rate |
| Ding et al [63]. | Tablet with an automated data transfer software, and a web-based clinical decision support app. | Weight | Bluetooth; daily | Clinical decision supported provided feedback on symptoms. |
| Guo, et al [62]. | Tablet, web-based app, a remote monitoring device | HF symptoms, medication intake, blood pressure, heart rate, and weight. | Manual entry; weekly | Remote consultations, electronic medical record viewing, and making appointments. |
| Hägglund et al [59]. | HIS consists of a tablet wirelessly connected to a weight scale, with specialized software | weight, HF symptoms, | Manual entry; daily | Tablet computers, preloaded with self-care education guidelines, medication plan, a visual analog scale for health performance feedback |
| Hayes et al [44]. | Beat-HF consists of a wireless telemonitoring system, connected to a scale | Weight | Bluetooth; daily | HF education |
| Koehler et al [49]. | A wireless telemonitoring system with a three-channel ECG, tablet, mobile phone, and telemonitoring-analysis software. | Weight, BP, HR, Oxygen saturation, EKG, and self-rated health status | Bluetooth; daily | self-care education |
| Koehler et al [50]. | PDA with Bluetooth functionality | EKG, BP, weight, self-assessed health status, | Bluetooth; daily | Pts. perform the daily self-assessment of health status by using the PDA interface. |
| LaFramboise et al [34]. | Portable telemonitor device (Health buddy) connected to the telephone line | Self-assessed HF symptoms | N/A | Pts. responded to HF symptoms questionnaire and received self-care education. |
| Lloyd et al [42]. | Tablet accessed application and an aerobic stepper | Medication intake, weight, and exercise | Manual entry; daily | Data entry, and watching HF video |
| Nundy et al [35]. | Smartphone | N/A | none | Participants received and responded to HF-related automated self-care education via short messages services |
| Pedone et al [55]. | Smartphone, transmitter | BP, weight oxygen saturation, HR | Via transmitter; heart rate and blood pressure: 2 x daily, Spo2: 3x daily, weight: daily | Smartphones provide activities reminders to the patients |
| Piotrowicz et al [60]. | Portable 3-lead EKG device, Smartphone | EKG, oxygen saturation, BP, HR, patient-reported symptoms | Bluetooth; continuously during exercise training; daily | Smartphones provided pre-exercise  symptoms  assessment |
| Radhakrishnan et al [37]. | Tablet, game application | N/A | N/A | Tablet provided a gaming interface |
| Rosen et al [41]. | Tablet, a specialized HF management protocol application | Weight, exercise, sodium intake, HF educational video | Bluetooth; daily | Tablet provided HF-related education interface |
| Seto et al [52]. | Smartphone with specialized HF symptom monitoring application, and web-based clinician and patient front-end | EKG, BP, weight, patient-reported symptoms | Bluetooth: daily, except EKG which was weekly | The mobile phone displayed symptom questionnaires, monitored parameters, feedback to the patients, Website displays monitored physiological information |
| Smeets et al [54]. | Smartphone with a specialized HF symptom monitoring application (CardioCoach app), and web-based clinician dashboard | BP, HR, weight, medications intake, and pt.-reported symptoms | Bluetooth; daily | Mobile phone for receiving notifications, and for HF education. The web dashboard displays secured information to clinicians. |
| Sohn et al [45]. | Fitbit Charge 2, Smartphone, web-based data integration platform | Physical activity, weight, medications | Bluetooth; continuously: physical activity; daily: weight and medications intake | Mobile phones display physical activity levels |
| Kitsiou et al [47]. | Fitbit Charge 2, mobile phone, web-based data integration platform(iCardia), App, (Health storyline) | Physical activity, weight, medication intake, BP. | Bluetooth; daily | Mobile phones display HF education. |
| Villani et al [56]. | PDA with a specialized app | Weight, BP, HR, BP, EKG, medications | Bluetooth, daily | PDA displayed symptoms’ questionnaires, anxiety, and depression visual scales. |
| Ware, et al[14]. | Smartphone with a specialized app | Weight, BP, HR, HF symptoms | Bluetooth; daily | A smartphone displayed feedback about transmitted parameters. |
| Wei et.al [49]. | Smartphone with HF management app | Weight, exercise, sodium intake | Bluetooth; daily | watching educational videos |
| Werhahn et al[52]. | mobile iOS-based applications, smartphones, smartwatches, tablet | Blood pressure and body weight, medication intake symptoms, daily step | Manually except daily steps and heart rate which were continuous. | Data acquisition and transmission |
| Zan et al[37]. | Tablet with a web platform for both patients and study investigators; Interactive voice response (IVR) system | Weight, BP, HR, daily | Bluetooth and interactive voice response system; daily | Tablet provided a graphic display of monitored parameters |
| Zhang et al[44]. | Smartphone app with virtual reality-based personalized self-care assistant | Weight, BP, HR; dietary sodium, medication, fluid intake, physical activity, HF symptoms; 3x daily | Interactive voice response system and manually | mobile phones provide an interface for an interactive virtual human assistant |
