# Supplementary material for: Measures of Engagement With mHealth Interventions in Patients With Heart Failure: Scoping Review
Source: JMIR Mhealth Uhealth. 2022 Aug 22;10(8):e35657. doi: 10.2196/35657 (PMC9446141; doi:10.2196/35657)
Supplement: Multimedia Appendix 4 [file mhealth_v10i8e35657_app4.docx]

Multimedia Appendix 4. The operational definitions of patient engagement with mHealth interventions

| Author | Operational Definition of Engagement | Subjective measure | Data collection method | Analytical methods |
| --- | --- | --- | --- | --- |
| Apergi et al [46]. | The percentage of the days that patient used technology to the number of days equipped with technology | N/A | N/A | Descriptive and inferential statistics |
| Athilingam et al [38]. | The number of days patients accessed mHealth app features; the proportion of patients that completed the daily measure of physiological parameters. | N/A | Usage logs per feature time stamped on a website | Descriptive and inferential statistics |
| Barlett et al [57]. | Time spent per mHealth device screen viewed (screen time); Number of days physiological parameters were measured during the possible days | ✓s | Logs of patients transmitted measures stored in webserver | Descriptive statistics |
| Buck et al [39]. | N/A | ✓s | N/A | Thematic analysis |
| Chow et al [61]. | Completion of ≥ 85% of the daily measure of physiological parameters and viewing 11 HF educational videos in the first 6 months | N/A | Not stated | Descriptive statistics |
| Dang et al [40]. | The percentage of days in 90 days that each patient responded to mobile phone administered symptoms questionnaires | N/A | Patients' responses stored in a webserver | Descriptive and inferential statistics |
| Deka et al [64]. | The number of video sessions completed from the required 8 sessions | N/A | Exercise diaries, Video session attendance  records kept on a paper copy | Descriptive statistics |
| Dendale et al [53]. | The percentage of daily measures completed | N/A | Not stated | Descriptive statistics |
| Ding et al [63]. | The number of days per week weight was monitored during the duration of the intervention. | N/A | Not stated | Descriptive statistics |
| Guo, et al [62]. | The number of pts. with 1 or more logins to the mobile or web app per week | ✓f | Cloud-based server | Descriptive statistics, and thematic analysis |
| Hägglund et al[60]. | Numbers of  days daily measures were completed, or educational videos viewed per number of days equipped with the system | N/A | Usage data is automatically captured and stored in the tablet | Descriptive statistics. |
| Hayes et al [44]. | Number of weight transmissions per patient per number of days without hospitalization or death during the study duration | N/A | Central server | Descriptive and inferential statistics |
| Kitsiou et al [47]. | Wear-time of Fitbit based on heart rate | N/A | Not stated | Descriptive Statistics |
| Koehler et al [49]. | Number of days per duration equipped with the system that the measured parameters were transmitted to the monitoring center | N/A | Not stated | Descriptive statistics |
| Koehler et al [50]. | Number of days patients transmitted measured parameters per day equipped with the system | N/A | Not stated | Descriptive statistics |
| LaFramboise et al [34]. | N/A | ✓f,✓s | Interviews with patients | Content analysis |
| Lloyd et al [42]. | The number of pts. that transmitted their measured parameters daily | N/A | Web-based server (RedCapp) | Descriptive and inferential statistics |
| Nundy et al [35]. | The numbers of SMS replies sent by patients in 30 days or numbers of SMS per day | N/A | Mobile phone SMS analysis | Descriptive statistics |
| Pedone et al [54]. | The percentage of daily monitored parameters completed by patients | N/A | Not stated | Descriptive statistics |
| Piotrowicz et al [60]. | The number of patients that completed and transmitted the daily pre-exercise symptom assessment and ECG. | N/A | Analysis of stored daily patients' responses and transmission of pre-exercise Questionnaire and ECG | Descriptive statistics |
| Radhakrishnan, et al [37]. | The average number of games played per patient, total game-playing time per patient | N/A | Not stated | Descriptive statistics to evaluate game playing time; the correlation between demographics, game playing time, and HF knowledge and self-care |
| Rosen et al [41]. | The percentage of the initial 120 days of the intervention that patients measured all monitored parameters | N/A | Not stated | Description statistics |
| Scherr et al [58]. | The percentage of physiological parameters measured and transmitted data per patient per effective monitoring days | N/A | Transmitted data was stored in a rational database and computed using a technical computing language, MATLAB, embedded in the system | Descriptive statistics |
| Seto et al [52]. | Average numbers of days per week that patients measured all daily monitored parameters | N/A | Transmitted parameters stored in a hospital data repository | Descriptive statistics |
| Smeets et al [54]. | The percentage of physiological parameters measured and transmitted data per patient per effective monitoring days | ✓f | Web-based server | Descriptive statistics, thematic analysis |
| Sohn et al [45]. | Fitbit: ratio of the number of hours a pt. has heart rate readings to the total hours in the study. Weight and medications: ratio of days patients have readings to total days in the study | N/A | Cloud-based servers | Descriptive statistics |
| Villani et al [56]. | Percentage of monitored parameters transmitted during the duration of the study | N/A | Transmitted parameters stored in the study’s server | Descriptive statistics |
| Ware et al. [14]. | Overall adherence was the proportion of days patients took all the daily monitored parameters per duration of enrollment in the stay. Incomplete adherence was the percentage of days patients took at least 1 reading but not all 4. | ✓s | Data extracted from the program web server | descriptive statistics: simple and multivariate regressions used to assess the effects of time, age, sex, and disease severity on adherence rates. |
| Wei et.al [48]. | The percentage of pts. that logged into the app at least daily or every other day; the number of logins per person. | ✓f | Not stated | Descriptive statistics |
| Werhahn et al [51]. | Percentage of the days that patient competed for measures of physiological parameters; Number of days in monitored days smartwatch was worn | N/A | Smartwatch usage data were extracted from the Clinical Document Architecture | Descriptive statistics |
| Zan et al [36]. | Percentage of the patients that logged in daily into the systems in the 3 months follow-up duration | ✓ | Patients' logins in the web portal of the app extracted from a web server | Descriptive statistics |
| Zhang et al [43]. | Not stated | ✓T, UGS | User Engagement Scale data and device built-in tracker | Descriptive statistics |

N/A: not reported; ✓f: Study used the semi-structured interview to describe engagement; ✓s: Study used focus groups to describe Patient engagement; ✓T: Study used think-aloud to describe patient engagement; UGA: Study used User Engagement scale to measure patient engagement.

Notes. 1. In studies in which no clear operational definition of engagement was provided. We derived the definitions based on the reported engagement outcome
