# Supplementary material for: Measures of Engagement With mHealth Interventions in Patients With Heart Failure: Scoping Review
Source: JMIR Mhealth Uhealth. 2022 Aug 22;10(8):e35657. doi: 10.2196/35657 (PMC9446141; doi:10.2196/35657)
Supplement: Multimedia Appendix 5 [file mhealth_v10i8e35657_app5.docx]

Multimedia Appendix 5. A Summary of descriptive characteristics of mHealth interventions engagement metrics, and reported outcomes of patient engagement with mHealth interventions

| Engagement characteristics | Number of studies n (%) |
| --- | --- |
| **Study design**  Randomized control trials (RCTs) [38, 47, 49, 50, 52, 53, 55, 56, 58-60, 63, 64]  Quasi experimental [35-37, 41, 42, 45, 51, 61]  Mixed method [14, 40, 43, 48, 54, 57, 62]  Secondary analysis of RCTs [44, 46]  Qualitative [34, 39] | 13(40.6)  8(25.0)  7(21.9)  2(6.3)  2(6.3) |
| **Type of intervention**  Telemonitoring [14, 36, 38-41, 43-63]  Self-care education [34, 35, 37]  Cardiac rehabilitation [60]  Self-care adherence without telemonitoring [42, 64] | 26(81.2)  3(9.4)  1(3.1)  2(6.3) |
| **Strategies for improving engagement** (number or articles, n = 13)  System generated alerts [34, 36, 41, 43, 45, 48, 54, 55]  Follow-up calls [43, 45, 49, 50, 52, 63]  System generated alerts and follow-up calls[53] | 7(53.8)  5(38.4)  1(7.8) |
| **Types of engagement metrics**  System usage data only[35-38, 41, 42, 44-47, 49-53, 55, 56, 58-61, 63, 64]  Qualitative measures only[34, 39]  System usage data and qualitative measures[14, 40, 48, 54, 57, 62]  System usage data, qualitative measures, and user engagement  questionnaires[43] | 23(71.8)  2(6.3)  6(18.8)  1(3.1) |
| **System usage metrics** (number of articles, n = 30)  Number of physiological parameters transmitted[14, 38, 42, 44, 45, 49-61, 63]  Number of heart failure questionnaires completed[40, 46]  Number of logins[36, 43, 48, 62]  Number of short message service (SMS) responses[35]  Time spent [37, 38, 45, 47, 48]  Number of features accessed, or screen viewed [57, 59, 61, 64] | 19(63.3)  2(6.7)  4(13.3)  1(3.3)  5(16.7)  4(13.3) |
| **Analytical approach to system usage data** (number of articles, n = 30)  Descriptive statistics only[35-37, 43, 45, 47-64]  Descriptive and inferential statistics [14, 38, 40, 42, 44, 46] | 24(80.0)  6(20.0) |
| **Engagement over time**  Reported[14, 36, 40, 41, 44, 46, 52, 62] | 8(26.7) |
| **Predictors of engagement**  Reported[14, 41, 45, 46] | 4(13.3) |
| **Effects of engagement on heart failure outcomes**  Reported[37, 42, 44, 45, 48, 57] | 6(20) |

Reported: A variable was assessed in the study otherwise not reported; n: the number of studies
