# Supplementary material for: Measures of Engagement With mHealth Interventions in Patients With Heart Failure: Scoping Review
Source: JMIR Mhealth Uhealth. 2022 Aug 22;10(8):e35657. doi: 10.2196/35657 (PMC9446141; doi:10.2196/35657)
Supplement: Multimedia Appendix 6 [file mhealth_v10i8e35657_app6.docx]

Multimedia Appendix 6. Reported outcomes of patient engagement with mHealth interventions

| Author | Reported Outcomes of Patient engagement | Effects of Engagement on study outcomes | Strengths | Limitations |
| --- | --- | --- | --- | --- |
| Apergi et al [46]. | Mean (SD): 35.3(26.0) and 37.8(28.9)times in 90 days in Alexa+ and Avatar groups respectively | N/A | Applied inferential statistics in examining the effects predictors of patient engagements | A small sample size, criteria for extraction, and selection of relevant time-stamped usage log were not described |
| Athilingam et al [38]. | Mean engagement was 78%; 44.4% (4/9) of pts. accessed the features daily and completed all required measures; 5/9 (55.5%) accessed the features over 80% (24 days) of the monitored period. | N/A | Provided objective measure of patient usage patterns | Applied only descriptive statistics in the analysis of usage logs. Small Sample size, criteria for extraction, and selection of relevant time-stamped usage log not described |
| Barlett et al [57]. | Blood pressure was measured on 84% of the possible days and weight on 88% of the days. Daily walks were less frequently recorded, on only 51% of the possible days. | Significant improvement in HF knowledge. | Used multiple methods to access the effect of engagement on HF outcomes. | Technical issues with connectivity or device hampered accurate record of data |
| Buck et al [39]. | 16.7 % (2/12) participants stated that they would continue to use the device every day | N/A | Rigorous qualitative analytical method | A small sample size, findings not generalizable, subjected to sociability bias |
| Chow et al [61]. | 74% (64/86) performed at ≥85% the daily measures; 65% (56/86) completed all 11 education videos. Mean: 69.8% | N/A | Included objective measure of engagement. | Lack of information about how engagement data were extracted and analyzed. The effect of engagement on clinical and patient-reported outcomes was not assessed |
| Dang et al [40]. | 73.8% (31 /42) used the system throughout the study duration of 3 months, range: (2–30) days, mean (SD)= 18.2(7.2) days; the total number of the usage times ranged from 21 to 87 days mean (SD): 62.0(15.9) | Assessed in interviews; the mHealth app made patients feel more secure about their health | Used multiple methods to access the effect of engagement on HF outcomes. | A small sample size, lack of standardized tools for usability evaluation |
| Deka et al [64]. | 78% of the participants completed 5 or more sessions. Overall session completion was 68% |  | Use both objective and subjective measures to collect data | A small sample size |
| Dendale et al [53]. | 83% of the required daily measures were transmitted; 76% of the HCP logged into the website at least once during the study. | N/A | Used objective measure | Incomplete information on how engagement data was extracted. |
| Ding et al [63]. | 45% (67/91), 74% (67/91) transmitted their weight 6 days/week, 4days/week of the monitored days, respectively. | N/A | Accounted for missed days of monitoring related to hospitalization, and situations beyond pts. control | Technical issue with connectivity |
| Guo et al [62]. | 66% (40/66) of the participants used the system ≥ once per week | N/A | low attrition rate 5% (4/70) | Analysis of engagement measures was limited to descriptive statistics |
| Hägglund et al [59]. | Median adherence of 88% [IQR: 78%, 96%] | N/A | objective measure | Did not correct for days spent in the hospital or abroad, at which times the system could not be used by the patient. Small sample size. Did not measure the duration of usage per day the system was used |
| Hayes et al [44]. | Reported adherence to daily weight transmission was 53.3% (47300/88751) person-days | 1-day increase in adherence was associated with a 19% decrease in the rate of death in the following week | Accounted for days pt. was hospitalized, or dead in the analysis; telemonitoring adherence was examined as a dynamic measure. | Nonadherence related to technical problems was not accounted for in the analysis. |
| Kitsiou et al [48]. | N/A | N/A | N/A | N/A |
| Koehler et al [49]. | 97% (743/765) of patients were at least 70% compliant with the daily transfer of data to the telemonitoring center. | N/A | objective measure; accounted for hospitalization days in the operationalization of engagement, large sample size | Incomplete information on how engagement data was extracted, |
| Koehler et al [50]. | 81% (287/354) were at least 70% compliant with the daily transmission of data | N/A |  |  |
| LaFramboise et al [34]. | Participants believed that prompts provided by the mHealth device increased their engagement with the device and HF self-care | Participants stated it improved their self-care, HF knowledge, and their overall health status | Provided an in-depth view of patients' experiences that may not be captured by objective measures. | Lack of generalization, small sample size, and subjected to sociability bias |
| Nundy et al [35]. | The response rate was 5.7 SMS (range 0-27) per duration of study or 1 SMS per 5 days, 33.3% (5/15) has a response rate of zero | N/A | Used an objective measure | A small sample size. |
| Pedone et al [55]. | Patients completed 62% of scheduled measurements; adherence for pulse oximeter was (70%), and weight (56%); 64% (32/50) completed ≥ 50% of the scheduled measurements. | N/A | Used an objective measure | Incomplete information on how engagement data was extracted, analysis of engagement data does not state |
| Piotrowicz et al [60]. | 100% (77) of patients in the home-based telemonitoring group responded and transmitted their daily assessment and ECG | N/A |  |  |
| Lloyd et al [42]. | 84% of the pts. completed the daily transfer of monitored parameters | Mean weight loss of 5.1lbs over the 30 days; increase in exercise duration at a rate of 0.08 min per day; 66% of the pts. adhered to medication (taking 75% of daily medications) | Applied both inferential and descriptive statistics | Small sample size |
| Radhakrishnan et al [37]. | The average number of times the game was played: 9.7 (range: 3–66): an average game-playing time (in minutes):237 (range: 12–1298) | Improvement in HF knowledge and HF self-management respectively. | An objective measure of duration and frequency of use | Small sample size; short duration of the study. |
| Rosen et al [41]. | median adherence: 96%; adherence |  | Low attrition rate 4% (2/50) | Small sample size; analysis limited to descriptive statistics. |
| Scherr et al [58]. | A median of 162 transmissions per patient (IQR 136-173) per effective monitoring day. Overall adherence: 95% (7554/ 7962) | N/A | Used effective monitoring days instead of total monitoring days to account for missed transmission related to factors outside patients' control | Relationship between engagement and HF outcomes not stated |
| Seto et al [52]. | Patients completed all the tasks in 5 to 6 days per week; 84% (42/50), 66%(33/50), and 32% (16/50) of the patients completed at least 91 (50%), 146 (80%), and 173 (95%) of monitored parameters respectively. | N/A | Used an objective measure, high drop attrition rate. | Engagement data might not be a representation of entire data transmitted daily by patients |
| Smeets et al [54]. | Pt completed 94.7% (4504/4758) of measured parameter transmissions. | Medication adherence was 92.6% | Used an objective measure, a low attrition rate of 4%; nonadherence related to technical issues accounted for. | Small sample size, high incidence of technical problems |
| Sohn et al [45]. | Median usage for the activity tracker was 79.1% for HR-hour and 75.4% for HR-minute. Medication and weight were 55%, and 59.7%, respectively. | Device usage negatively correlated with changes in self-care confidence; HF quality of life was not correlated with age, gender, EF, NYHA class, or education | Accounted for hospitalization when patients could not transmit data | A small sample size. Patients shared scale with family members, lack of a rigorous method to verify weight readings. |
| Villani et al [56]. | 80% of the required monitored parameter were transmitted by patients | N/A | Objective measure, the low attrition rate | A small sample size: lacked detailed analysis of engagement data, such as changes in engagement over time. |
| Ware et al [14]. | The overall average engagement was 73.6% (SD: 25.0), | Patients stated that engagement in telemonitoring was associated with improvement in HF self-care | An objective measure of the change in engagement over time. Used both objective and subjective measures of engagement. | No information about the attrition rate |
| Werhahn et al [51]. | The engagement was 82.95% for blood pressure and 78.18% for body weight; smartwatch 587/692 (84.8%) wear days per monitored day; mean daily wearing time was 12.71 ± 1.13 h. | N/A | An objective measure of change in engagement over time. Used both objective and subjective measures of engagement. | questionnaires were not standardized, a small sample size |
| Wei et al [48]. | 70% (7/10) logged into the app every other day, while 50(5/10) logged into the app daily; the average number of logins per person was 403, duration per session was 5 mins | Increased app use correlated with improvement in heart failure knowledge and quality of life and weight loss | A low attrition rate in the intervention group, 16.7% (2/12) | Reported only descriptive statistics, small sample size, and short duration of the study |
| Zan et al[36]. | More than 50% of the patients logged in daily in 72/90 (80%) follow-up days | 7 (33.3%) patients stated that engagement in telemonitoring was associated with improvement in HF self-care | Used subjective measures of engagement. | small sample size, analysis limited to descriptive statistics |
| Zhang et al [43]. | not stated | N/A | objective measure | A small sample size, study protocol |
